# Prevalence of multi drug resistant *Acinetobacter baumannii* in the clinical samples from Tertiary Care Hospital in Islamabad, Pakistan

**DOI:** 10.12669/pjms.295.3695

**Published:** 2013

**Authors:** Shahzeera Begum, Fariha Hasan, Shagufta Hussain, Aamer Ali Shah

**Affiliations:** 1 Shahzeera Begum, Department of Microbiology, Faculty of Biological Sciences, Quaid-i-Azam University, Islamabad, Pakistan.; 2 Fariha Hasan, Department of Microbiology, Faculty of Biological Sciences, Quaid-i-Azam University, Islamabad, Pakistan.; 3 Shagufta Hussain, nMicrobiology Laboratory, Pakistan Institute of Medical Sciences, Islamabad, Pakistan.; 4 Aamer Ali Shah, Department of Microbiology, Faculty of Biological Sciences, Quaid-i-Azam University, Islamabad, Pakistan.

**Keywords:** *Acinetobacter baumannii*, AmpC β-lactamases, Carbapenemases, Extended-spectrum β-lactamases, Multi-drug resistant, Minimal inhibitory concentration

## Abstract

***Background & Objectives:*** *Acinetobacter baumannii* can cause a wide range of infections, including bacteremia, pneumonia, urinary tract infection, peritonitis, etc. This organism is becoming resistant to a large group of antibiotics, especially β-lactam antibiotics. The reason for multi-drug resistance may be the production of extended- spectrum β-lactamses (ESBLs), carbapenemases/metallo β-lactamases or AmpC β-lactamases. The aim of the present study was to determine the prevalence of multi-drug resistant *Acinetobacter* *baumannii* isolated from the patients in Surgical Intensive Care Units (SICUs) of Pakistan Institute of Medical Sciences (PIMS), Islamabad, Pakistan.

***Methods:*** A total of 91 *A. baumanni* isolates were collected from PIMS during the period from February 2011 to December 2011. The antibiotic susceptibility testing was performed by standard disc diffusion method as recommended by CLSI. Combination disc method, Modified Hodge test, EDTA disc synergy test and AmpC disc test were performed for detection of ESBLs, carbapenemases, metallo β-lactamases, and AmpC β-lactamases, respectively.

***Results:*** The prevalence of MDRs was reported 100% among *A. baumannii*. The antibiotic susceptibility profile showed that minocycline and tigecycline were the most effective drugs against *A. baumannii.* Almost all of *A. baumannii* isolates were carbapenemase and metallo β-lactamase producers. AmpC prevalence was observed in 41.76%, while none of the isolates was ESBL producer. Antibiogram and minimal inhibitory concentrations (MICs) indicated tetracycline is relatively effective against *A. baumanii*.

***Conclusions:*** Increased frequency of multi-drug resistance supports the need for continuous surveillance to determine prevalence and evolution of these enzymes in Pakistan.

## INTRODUCTION

Hospital-acquired infections are a major challenge to patient safety. It is estimated that, a total of 1.7 million hospital-acquired infections occurred (4.5 per 100 admissions every year), and almost 99,000 deaths were associated with a hospital-acquired infection, making hospital-acquired infections the sixth leading cause of death in the United States Hospital-acquired infections are most commonly associated with invasive medical devices or surgical procedures.^1^ Recent data from the U.S. National Healthcare Safety Network indicate that gram-negative bacteria are responsible for more than 30% of hospital-acquired infections, and these bacteria predominate in cases of ventilator-associated pneumonia (47%) and urinary tract infections (45%)**. **In intensive care units (ICUs) in the United States, gram-negative bacteria account for about 70% of these types of infections, and similar data are reported from other parts of the world.^2^

Global data reveals that multidrug-resistant *A. baumannii* is emerging as a common hospital-and community-acquired infection that is difficult to treat. It is a very resistant and aggressive organism that infects patients with weakened defenses like ICU patients and those with invasive devices.^3^ In large surveillance studies from the United States, between 5 and 10% of cases of ICU-acquired pneumonia were due to *A. baumannii. A. baumannii* may occasionally cause skin/soft tissue infections outside of the military population. The organism caused 2.1% of ICU-acquired skin/soft tissue infections in one assessment. *A. baumannii* is an occasional cause of UTI, being responsible for just 1.6% of ICU-acquired UTIs in one study.* A. baumannii* has been reported to be a more common cause of ICU-acquired bloodstream infection than of non-ICU-ward infection (1.6% versus 0.9% of bloodstream infections, respectively, in those locations). Crude mortality overall from *A. baumannii* bloodstream infection was 34.0% to 43.4% in the ICU and 16.3% outside the ICU.^4^

This organism has multiple mechanisms for resistance including an impermeable outer membrane, enzymes which breakdown of antibiotics especially AmpC β-lactamases, class D OXA-type and class B metallo-β-lactamases which allow the organism to resist carbapenems, porin channels alterations as well as efflux pumps, and other genetic changes that may lead to resistance to fluoroquinolones.^5^ All *A. baumannii* strains are chromosomally encoded AmpC cephalosporinases also known as Acinetobacter-derived cephalosporinases (ADCs). Extended-spectrum β-lactamases (ESBLs) from the Ambler class A group have also been described for *A. baumannii*, but assessment of their true prevalence is hindered by difficulties with laboratory detection, especially in the presence of an AmpC. More recent focus has been on VEB-1, which disseminated throughout hospitals in France (clonal dissemination) and was also recently reported from Belgium and Argentina (VEB-1a). Other ESBLs identiﬁed in *A. baumannii* include TEM-92 and -116 from Italy and The Netherlands, respectively, and SHV-12 from China and The Netherlands. Also, CTX-M-2 and CTX-M-43 have been described from Japan and Bolivia, respectively.^6^

In the present study, multi-drug resistant *Acinetobacter baumanii* were isolated from hospitalized patients and reasons to their resistant to beta-lactam antibiotics were investigated.

## METHODS

The present study was jointly conducted by the Department of Microbiology, Quaid-i-Azam University, Islamabad and Department of Pathology, Pakistan Institute of Medical Sciences (PIMS), Islamabad, during the period of February-December 2011. This hospital is the prime tertiary care facility in the public sector for Islamabad and its surrounding population.

***Samples: ***A total of 91 *Acinetobacter baumannii* strains were isolated from the wound samples collected from the patients in out-door patients department as well as admitted in ICUs, medical and surgical wards of Pakistan Institute of Medical Sciences, Islamabad. These pathological samples were preceded for isolation and identification according to standard microbiological laboratory methods.

***Media, Reagents & Kits: ***MacConkey and blood agar (Oxoid Ltd., Cambridge, UK) medium were used during the processing of isolates. MacConkey agar medium helps in the differentiation of lactose fermenters and non- fermenters. Blood agar medium helps in the differentiation of hemolytic and non-hemolytic colonies. Iso-Sensitest agar medium (Oxoid Ltd., Cambridge, UK) is used for the determination of resistance pattern against different antibiotics groups. Isolates were identified by API 20 E kit (Biomeriuex, USA). Analytical profile index (API) is a standardized identification system for Enterobacteriaceae.

***Antibiotics: ***Following antibiotics (Oxoid Ltd., Cambridge, UK) were used for antimicrobial susceptibility testing against *A. baumannii*, supplied by Oxoid Ltd., Basingstoke, UK: amoxacillin- clavulanic acid 20/10 µg, piperacillin 100 µg, ceftazidime 30 µg, meropenem 10 µg, gentamicin 10 µg, amikacin 30 µg, tetracycline 30 µg, ciprofloxacin 5 µg, tigecyclice 15 µg, minocycline 30 µg, imipenem 10 µg, cefotaxime 30 µg, doxcycline 30 µg, polymyxin B 10 µg, tobramycin 10 µg, ampicillin-sulbactam 10/10 µg, pipercillin- tazobactam 100/10 µg, trimithoprim-sulfamethoxazole 1.25/23.75 µg.

***Identification of bacterial isolates: ***The isolates were identified and characterized using standard microbiological methods like colony morphology and Gram’s staining. API 20 E kit (Biomeriuex, USA) was also used for identification purpose.

***Determination of antibiotic resistance patterns of Acinetobacter baumannii: ***Antibiotic resistance patterns of *A. baumannii* isolates against different groups of antibiotics was determined by employing disc diffusion by Kirby-Bauer method. Bacteria were classified as susceptible, intermediate or resistant to antibiotics in accordance with current Clinical Laboratory Standard Institute (CLSI) recommendations. The prevalence of multi-drug resistant *A. baumannii* was checked in different specimens, wards as well as in the patients from different age groups.

***Phenotypic tests for the detection β-lactamases production by multi-drug resistant A. baumanii:***

Double-disc diffusion test was used to determine the ESBLs producer as reported by Sirot in 1996.^7^ Carbapenamases production was confirmed by Modified Hodge Test, Lee et al^8^ protocol was followed for metallo β-lactamases producers. AmpC disk method as explained by Black et al^9^ was used for the determination of AmpC β-lactamases producers.

***Minimum Inhibitory Concentration (MIC): ***Agar dilution method was used to determine the MICs of ciprofloxacin and tetracycline. Standard powders of antibiotics were used to make stock solutions. Antibiotic dilution range of 0, 1, 2, 4, 8, 16, 32, 64, 128, 256, 512, 1024 µg/ml was prepared in flasks according to the antibiotic breakpoint for the particular species. No antibiotic was added to control. 50 ml of cooled molten agar was added to each flask, including the antibiotic free control. Mixed well and poured into the 150 mm perti dish, allowed agar to set and then dry surface of plates and used immediately**. **Four colonies of isolates were transferred to nutrient broth and placed in incubator shaker at 35-36°C until the visible turbidity was equal to 0.5 McFarland standards. Multipoint inoculators were used to deliver 1-2 µl of suspension on to the surface of the agar. The plates were then incubated at 35°C and growth was checked at different concentrations of antibiotics.

**Fig.1:**
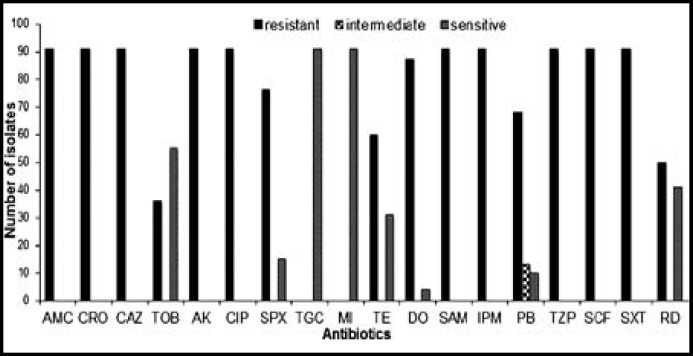
Antibiotic susceptibility profile of multi-drug resistant *A. baumannii*

**Fig.2:**
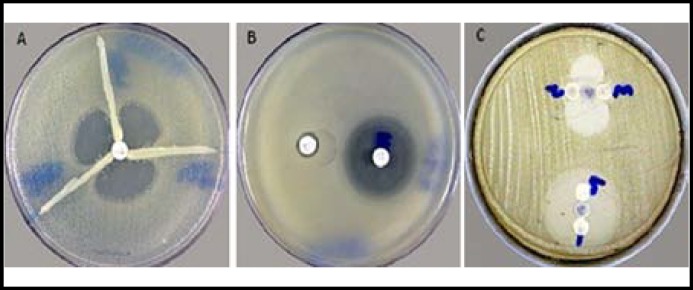
Positive result for the different β-lactamases producing MDR *A. baumannii* strains. (a) Carbapenamase detection test, (b) MBL detection, (c) AmpC detection

**Fig.3:**
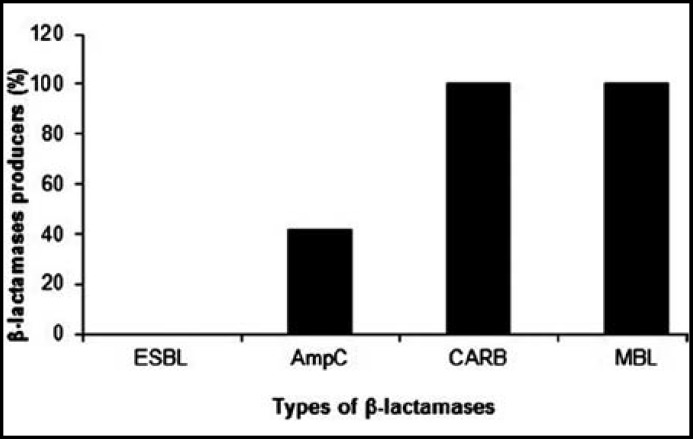
Prevalence of different β-lactamase producing *A. baumannii*

**Fig.4:**
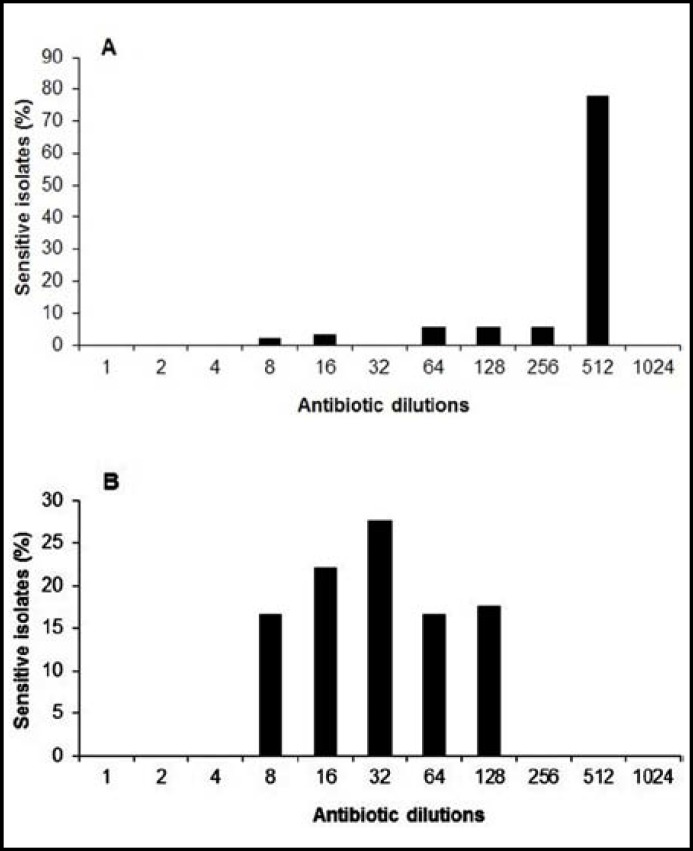
Mininmal inhibitory concentrations of (a) ciprofloxacin (512 µg/ml) and (b) tetracycline (128 µg/ml) for the MDR *A. baumannii*

## RESULTS

A total of 91 bacterial isolates were collected from the indoor and outdoor patients who visited or were admitted at PIMS and identified on the basis of colony morphology and biochemical tests.

***Identification of isolates: ***Isolated colonies on Blood and MacConkey agar were used to study colony characteristics. On MacConkey agar colonies of *A. baumannii* appeared as non-lactose fermenter and on blood agar colonies were about 1 to 2 mm in diameter, non-pigmented, domed, and muciod, with smooth to pitted surfaces.* A. baumannii* were oxidase negative and non motile. The isolates were identified as *Acinetobacter baumannii *by using API 20 E kit.

***Antibiotic resistance pattern of A. baumannii:*** *A. baumannii* exhibited the highest resistance (91, 100%) against cephalosporins, carbapenems, flouroquinolones and β-lactam drugs. Among aminoglycosides, tobramycin showed better activity than amikacin. Tetracycline also showed highest resistance (60, 65.93%) while tigecycline and minocycline showed zero resistance (91, 0.00%). Among all antibiotics used in this study, tigecycline and minocycline were found to be most effective against *A. baumannii *(Fig.1). All the clinical isolates of *A. baumannii* were found resistant to most of the antibiotics, and were considered as multi-drug resistant.

***Prevalence of multi-drug resistant A. baumannii in different specimens: ***Among the 91 samples, the highest prevalence of *A. baumannii* was observed in the endotracheal tubes secretions (23, 25.27%) followed by tracheal secretions (18, 19.78%) and and the least in pus (15, 16.48%).

***Prevalence of multi drug resistant A. baumannii in different wards: ***Highest prevalence of *A. baumannii* was found in neo-natal intensive care unit NICU (37, 42.85%), followed by Medical (ICU) (18, 19.78%) and the least in out-patient department (9, 9.89%).

***Prevalence of muti-drug resistant A. baumannii in patients of different age-groups: ***Prevalence of *A. baumannii* was found to be higher in new born babies as compared to the younger or older patients. Among 91 patients infected with *A. baumannii*, the highest percentage belonged to the age-group between 0-29 days (37,42.85%) followed by age groups between 40-60 years (17, 18.68%) and age groups between 1-20 years (14, 15.38%).

***Different β-lactamases producing multi-drug resistant A. baumanii: ***Among the 91 cephalosporin resistant isolates of *A. baumannii*, none was found to be ESBL positive. All the 91 carbapenem resistant *A. baumannii* isolates, were found to be carbapenemase producers (Fig.2a) and all of them were found to be metallo-β-lactamase producers (Fig.2c). All the 91 *A. baumannii* isolates were resistant to cefoxitin. AmpC β-lactamase detection showed that 21 (23.07%) isolates were strongly positive, 17 (18.68%) isolates were weakly positive and 53 (58.24%) isolates were negative (Fig.2c). *A. baumannii* were producing different kinds of β-lactamases (Fig.3).

***MIC Determination:*** MICs of ciprofloxacin and tetracycline were determined. MIC of ciprofloxacin was ranging from 64 to ≥512 μg/ml and tetracycline from 8 to 128 μg/ml. Both the antibiotics inhibited the growth of inoculated isolates at different dilutions (Fig.4).

## DISCUSSION

In the present study *A. baumannii* was found to be most frequent cause of nosocomial infections. In the present study, infections due to *A. baumannii* were found to be more prevalent among newborns than adults. *Acinetobacter *spp. have emerged as particularly important organisms in intensive care units (ICUs), and this is probably related, at least in part, to the increasingly invasive diagnostic and therapeutic procedures used in hospital ICUs in recent years.^10^ The proportion of newborns to adults’ patients varies from study to study and area to area. The prevalence of infections due to *A. baumannii* was higher in the newborn as compared to young and very old patients.

In our study, the highest prevalence of infections due to *A. baumannii* was observed in the ETT (endotracheal tube) specimens followed by treacheal secretions, and pus. Shanthi and Sekar^10^ reported that most of the isolates of *A. baumannii* were obtained from the respiratory tract (41.8%) followed by urinary tract (25.5%), wound (20%) and blood (12.7%). The highest prevalence of infections was observed in the NICU (42.85%), followed by medical ICUs (19.78%) and emergency (9.89%). 

The resistance patterns of *A. baumannii* towards various antimicrobial agents were determined by disc diffusion method. In the present study, *A. baumannii* exhibited the highest resistance 100% against cephalosporins, carbapanems, β-lactam inhibitors. The specific issue of *in vitro* testing of β-lactamase inhibitor combinations has been assessed by Higgins et al.^11^ They showed that the *in vitro* results for β-lactamase inhibitor combinations against *A. baumannii* are determined mainly by the activity of the inhibitors alone and therefore influenced by whether a fixed ratio of β-lactam to inhibitor or a fixed concentration of inhibitor is used Among aminoglycosides, amikacin showed 100% resistance, while tobramycin was found to be effective against *A. baumannii* with (39.6%) resistance. Among fluoroquinolones, ciprofloxacin showed more resistance as compared to sparfloxacin.

Disc diffusion should also be performed using Mueller-Hinton agar. Swenson et al^12^ assessed these CLSI-recommended methods and identified several problems in testing β-lactam antibiotics. First, very small colonies or a star-like growth was frequently observed in wells containing high concentrations of β-lactam antibiotics. This apparent growth beyond a more obvious end point makes determining an MIC by broth microdilution methods quite difficult. Second, there were many discrepancies between results obtained by broth microdilution and those obtained by disc diffusion. In contrast to the findings with these β-lactams, there was little MIC and zone diameter discrepancy for carbapenems, aminoglycosides, fluoroquinolones, and trimethoprim-sulfamethoxazole.

The transferable nature of ESBL in *Acinetobacter* may lead to increase in the basal level of multi- drug resistance among other nosocomial pathogens by dissemination and integration of the R-plasmids.^13^ Mahua et al^14^ reported a high incidence of ESBL production (28%) in *A. baumannii*. Aubert et al^15^ and Walther-Rasmussen and Hoiby^16^ reported Class D OXA β-lactamases are usually robust penicillinases (oxacillinases). Some OXAs (i.e., OXA ESBLs) are also able to hydrolyze extended-spectrum cephalosporin. In our study no single isolate was found to be ESBL producer.

Thus far, carbapenems have been thought of as the agents of choice for serious *A. baumannii* infections. However, although these drugs are still active against the vast majority of *A. baumannii* strains worldwide, the clinical utility of this class of antimicrobial is increasingly being jeopardized by the emergence of both enzymatic and membrane-based mechanisms of resistance^16^. The increase in the number of MBLs in *A. baumannii* is an ominous development in the global emergence of resistance to β-lactams. Rasmussen and Bush^17^ reported, carbapenem resistance can be due to acquired carbapenemase production. Carbapenem resistance is mainly due to either reduced levels of drug accumulation or increased expression levels of the pump efflux. Other risk factors responsible for colonization and infection with MBL producers include age of patient, duration of hospitalization, underlying diseases like diabetes, tumours or overcrowding in the hospital wards.^10^ The frequency of AmpC β-lactamase producing *A. baumannii* isolates in our study was 41.75%. Heritier et al^18^ reported that *Acinetobacters* may develop resistance to carbapenems through various combined mechanisms, including AmpC stable depression, decreased permeability, altered penicillin-binding proteins (PBPs) and, rarely, efflux pump over expression. 

## CONCLUSION

The primary goals for the control of multidrug resistant *Acinetobacter* infection are recognizing its presence in a hospital or long-term care facility at an early stage, controlling spread aggressively, and preventing the establishment of endemic strains. Control measures are based almost entirely on experiences from outbreaks of *Acinetobacter* infection and generally address the organism’s major epidemic modes of transmission and the excessive use of broad-spectrum antibiotics.

## Notes

***Source of funding:*** University Research Fund.
